# Artificial intelligence and breast cancer screening in Serbia: a dual-perspective qualitative study among radiologists and screening-aged women

**DOI:** 10.3389/fradi.2026.1714029

**Published:** 2026-02-24

**Authors:** Sofija Jovanović, Jelena Vukićević, Biljana Kilibarda, Marko Milosavljević, Vesna Bjegović-Mikanović

**Affiliations:** 1 Center for Radiology, University Clinical Center of Serbia, Belgrade, Serbia; 2 Institute of Ethnography of the Serbian Academy of Sciences and Arts, Belgrade, Serbia; 3 Institute of Public Health of Serbia “Dr Milan Jovanović Batut”, Belgrade, Serbia; 4 Institute of Social Medicine, University of Belgrade, Faculty of Medicine, Belgrade, Serbia

**Keywords:** artificial intelligence, breast cancer screening, focus groups, mammography, qualitative research

## Abstract

**Background:**

Breast cancer screening (BCS) by mammography was introduced globally in the last decades of the previous century and has been implemented in opportunistic or population-based models worldwide ever since. In Serbia, the national BCS Program was established in late 2012. Despite its existing framework, the Program's coverage remains suboptimal, and novel approaches to its optimization are being explored. The increasing use of artificial intelligence (AI) technology in numerous fields has been a hallmark of the previous decade, with AI-based solutions in breast imaging at the forefront of many research initiatives. Qualitative research has been previously conducted from Australia to Sweden, yielding insights into the AI-radiologist interaction, as well as the acceptability of screening-aged women toward AI use in screening. This study aims to gauge the stakeholders’ perspectives—radiologists’ and women's—on AI use in BCS in Serbia and help inform policy adaptations to maximize the prospective effectiveness of this public health intervention.

**Methods:**

Four focus groups (FGs) were organized in total, two with radiologists and two with screening-aged women, in Belgrade and Novi Sad. Residents in training and radiology specialists were divided for maximal discussion liberty. Two research members analyzed the discussion transcripts using a mixed inductive-deductive approach with a flexible coding frame.

**Results:**

Radiologists in this study see room for and have an overall cautiously positive attitude toward the application of AI in mammography BCS in the future. If AI were to perceptibly improve the current state of healthcare, such use of AI could be met with support among BCS-aged women.

**Conclusions:**

This study represents the first step towards understanding the attitudes of radiologists and screening-aged women in Serbia towards the use of AI in mammography. Additional studies will be necessary to get a more comprehensive overview.

## 1 Introduction

Breast cancer is the most common malignancy among women, both worldwide and in the Republic of Serbia, and as such presents a prevailing public health challenge on a global and national scale (1–4). While treatment outcomes and survival rates have improved significantly, the incidence of breast cancer continues to increase in both developed and developing countries. In Serbia, there is a consistent annual increase in the standardized incidence rate, making it a leading cause of cancer-related mortality among women (3).

To mitigate the incidence of advanced-stage cancers and overall disease burden, breast cancer screening (BCS) was globally introduced in the last decades of the previous century as a preventive measure within the population of healthy women (5). Depending on the country's healthcare legislation, BCS is implemented in opportunistic or population-based screening models (6). For population-based models, according to acknowledged guidelines by the European Society of Breast Imaging (EUSOBI), mammography at two-year intervals remains the reference screening standard (7, 8).

The increasing use of artificial intelligence (AI) technology in numerous fields of study has been a technological hallmark of the previous decade (9). Among the many applications machine learning (ML), and its extension, deep learning (DL), have found in medicine, the most notable strides have been made in the subfield of medical imaging (10). Because of its prevalent pathology and the abundance of imaging modalities available, AI-based solutions in breast imaging have found themselves at the forefront of the research initiative (11–13). Its application in screening mammography, in particular, remains a focus point in the scientific community, owing partly to the large publicly available datasets for model training (14, 15, 121). A number of possible uses have been examined in BCS, from pre-screening identification of high-risk individuals for targeted outreach, to examples of AI tools for mammography proper for breast mass detection, segmentation, and classification, across post-acquisition image quality improvement, all the way to future prediction of disease (14, 16–20). To increase effective BCS coverage, their points of integration into the BCS mammography workflow have also been analyzed in depth. There are numerous solutions, including AI-assisted image acquisition in decentralized sites, AI triage to reduce radiologist workload, and hybrid workflows (i.e., single-reader + AI) that approximate double-reading, among the most widely suggested potential solutions for settings facing a radiology workforce gap, as is the case in Serbia (2, 21, 22).

Qualitative analysis (QA) is a methodological approach for describing textual data in various forms: written, oral, or visual (23–28). This analysis approach serves to uncover both the manifest and latent, contextually-inured meaning through the creation of categories as parts of the coding frame to describe the researched phenomenon by abstraction, structuring, and classification of data (23–27, 29–31). The two most commonly employed QA data collection strategies are FGs and in-depth interviews (32).

In the Republic of Serbia, the national program for breast cancer prevention (hereafter referred to as the Program) was established in late 2012, marking the country's transition from opportunistic to population-based BCS (33). It constitutes a biennial, decentralized screening scheme for women aged 50–69 years and has been running continuously since, in two-year cycles. The Program incorporates target population identification, invitation, scheduling, examination, and reporting. Screening mammography is performed in two standard projections, and the subsequent mammograms are interpreted independently by two radiologists, who each yield one BI-RADS (abbr. Breast Imaging Reporting and Data System) score per breast. In case of any score divergence, a third radiologist is introduced for arbitration purposes (34). As of January 2025, the seventh screening cycle is underway.

Despite the existing national framework and allocated resources, BCS coverage in Serbia has been suboptimal in the past (2, 33, 35). Given the distribution of reported BCS coverages in Europe and especially the region, this may not be an unusual finding, but it has extended an open invitation to the country's policy-makers on the topic of Program improvement (36, 37). In response to this, the Ministry of Health of the Republic of Serbia has launched the Healthcare Academy in November 2024, a pilot collaboration with Sweden—the exemplar for organized BCS in Europe and the world—with the aim of Program optimization, as well as updated the comprehensive Guidelines on breast cancer clinical practice in 2025 (38, 39). Novel approaches to Program optimization are currently being explored.

In public health research, QA serves as a tool to explore complex interventions on an individual or community level, which can help guide policy-makers in anticipating and addressing potential challenges in their implementation down the line (40, 41). In the context of AI integration in mammography screening, QA constitutes a lens for understanding the real-world complexities of its introduction into the BCS workflow from up close for both stakeholders' points of view. In addition to population-based, quantitative surveys on the topic—the results of which have been published in abundance—QA-based research has been previously conducted worldwide from Australia to Sweden, yielding insights into the AI-radiologist interaction, as well as the acceptability of women undergoing BCS toward AI use in screening (42–48).

Hence, the aim of this study was to gauge the two stakeholders' perspectives—radiologists' and women's—toward BCS and the introduction of AI into screening workflows, including their understanding, expectations, and trust, in order to help inform future policy adaptations within the BCS Program in Serbia.

## 2 Methods

### 2.1 Study design

This is a qualitative, exploratory investigation of stakeholder knowledge levels, opinions, and practices regarding mammography BCS and the use of AI-based tools therein. It represents the first part of a larger, three-phase, mixed-method research effort on the subject. Cross-sectional by design, the study employs the thematic analysis method to examine and elucidate the items in question from the perspectives of healthcare providers (radiologists) and healthcare users (women) in BCS.

### 2.2 Setting and participants

Four focus groups (FGs) were organized in total.

Two FGs were organized with the healthcare providers' (radiologists’) stakeholder subset. The subset was further divided into radiology specialists and radiology residents in training. Each FG was entirely comprised of either specialists or residents to give participants maximum liberty to share their respective viewpoints. The radiologists' FGs took place at a designated conference room at the Institute of Public Health of Serbia “Dr. Milan Jovanović Batut” (IPHOS), with a U-shaped table layout to facilitate open discussion and direct interaction among participants.

Two FGs were organized with screening-aged women. To ensure geographic and experiential diversity, they were held in two separate cities (Belgrade and Novi Sad) at the headquarters of a contracted external public opinion research agency. Each session took place in a dedicated meeting room equipped with a round table to provide a neutral, comfortable environment conducive to discussion.

### 2.3 Sampling, discussion duration, and transcription

Participants were recruited using non-probability, purposive sampling strategies tailored to each stakeholder (sub)set.

Radiology specialists were recruited through criterion-based sampling with convenience access, at a two-day continuing medical education workshop on screening mammography at the IPHOS from 10 to 11 July 2024. All registered attendees (38) were invited to participate in the study, and those who volunteered (9) formed the specialist FG. The FG took place on the afternoon of the first workshop day and lasted approximately 90 min.

Radiology residents were recruited via the snowball sampling method. A radiology resident affiliated with the Cancer Screening Office at IPHOS, who served as a study liaison, initiated the recruitment during a didactic session as part of the radiology residency curriculum. Interested colleagues were invited to share their contact information, after which email invitations (12) containing a brief summary of the study were sent out. Those who responded positively and agreed to participate (7) formed the resident FG. The FG took place the following week, on a date chosen to cater to the availability of the majority (15 July 2024), and lasted approximately 120 min.

Screening-aged women were recruited by criterion-based purposive sampling with maximum variation, facilitated by an external public opinion research agency. The primary inclusion criterion was age (50–69 years), following the target demographic of the national BCS program. The key exclusion criterion was a confirmed history of breast cancer, due to the sensitive nature of the topic and the potential for such experiences to dominate or unintentionally constrain group discussion. The sample was deliberately heterogenous and balanced: approximately half of the participants had previously undergone a mammographic examination, while the other half had not. This intentional distribution was designed to spark richer conversations by introducing a variety of perspectives and experiences related to BCS. The first FG featured nine participants, while the second included eight, and they took place on 14 and 15 August 2024, respectively. The discussions lasted approximately 90–120 min each.

The FGs were video-recorded, and the recordings transcribed verbatim by the external public opinion research agency. The agency's role was limited to participant recruitment logistics (for 2 FGs), provision of facilities (for 2 FGs), recording equipment and verbatim transcription (for all FGs); it had no involvement in study design, FG moderation, data analysis or interpretation. Two researchers performed revision of the original audio-visual files to verify transcription accuracy, and the files were subsequently deleted.

### 2.4 Discussion guides

Separate semi-structured discussion guides were developed for radiologists and women, constructed on both a literature review of the matter and new investigative insight.

The authors performed a PubMed search to familiarize themselves with the discourse and evidence on artificial intelligence applications in BCS. Engineers from the Institute for Artificial Intelligence of Serbia with relevant expertise were subsequently contacted and invited to share their insights. Based on prior experience with a diagnostic mammography AI-based classification model, two engineers were recruited for semi-structured interviews. The interviews were conducted in an online setting (via Microsoft Teams) and lasted approximately one hour each.

A brief inductive QA of the interview content was performed, and the main themes (“specific software needs and expectations” and “radiologists’ digital literacy”) and most frequently used phrases were identified. These results and the literature review notes were then used to create a discussion guide for the radiologists' FG. Based on the literature review, a separate discussion guide was put together for the women's FGs.

After the initial development process, the two guides were tested through pilot interviews: the healthcare provider-aimed guide with a medical doctor, a member of the Cancer Screening Office at IPHOS, and the healthcare user-aimed guide with a screening-aged volunteer. For maximum clarity, slight semantic adjustments were made during this step. The discussion guides were then forwarded to all research team members for commentary.

The final versions of each guide began with a short introductory section and an ice-breaker. For radiologists, this included name, place of employment, training status (resident or specialist), and years of experience in radiology and/or mammography. For women, the ice-breaker covered name, age, vocation, a self-selected personal detail, and a brief reflection on how they were feeling at the time of the discussion.

The remainder of the radiologist guide consisted of the following:

- a general exploratory section on AI (covering participants’ understanding of it and its current and potential applications in medicine),
- a section on BCS (including definitions of screening, existing organized screening programs, prior experience working in screening, and perceptions of screening practice in Serbia),
- and the final section focused on the application of AI in BCS, specifically (exploring anticipated future uses, emotional responses to AI integration, perceived benefits and barriers, and reflections on professional identity and career choice in light of the emerging AI technologies).

The women's guide contained the following:

- a section on BCS (inquiring into the definition of screening, prior experience with mammography, reasons for deferring or not attending BCS, motivations influencing participation, and perceptions of breast cancer risk),
- a section on the use of AI in BCS (understanding of the mammography reading process as currently performed, opinions on AI involvement, acceptable and unacceptable use scenarios, examples of hybrid reading, and perceived barriers to implementation).

The FGs were moderated by one of the authors, a facilitator trained in QA methodology, who was not involved in the participants' clinical care or professional supervision. Several other measures were taken to mitigate bias and facilitator influence, and to maintain balanced power dynamics. The facilitator did not disclose their affiliation, outside of their researcher role, and used neutral prompts. The FGs with women, as healthcare users, took place in the external research agency's headquarters. Separate discussion guides were used for women and radiologists, with open-ended, non-leading questions ordered from general to specific. Women's groups were purposively composed to include participants with and without prior screening experience, and a breast cancer diagnosis was established as an exclusion factor in the recruitment phase, while the radiologists were grouped by training level to limit hierarchical influence. Ice-breaker questions and active moderation were used to encourage balanced participation.

### 2.5 Data analysis

Given the guide structure, discussion dynamic, and nature of the transcribed content, a mixed (inductive-deductive) QA method was employed. Two research team members independently read the material repeatedly to identify key points and preliminary categories and develop a comprehensive understanding of them.

A preliminary coding frame was developed based on both the literature and the discussion guides. The material was divided into coding units. Several rounds of manual coding by both authors ensued to test the coding frame, during which discrepancies were discussed and resolved by consensus to ensure consistency. Following the line of analysis, the coding frame was evaluated for internal coherence and thematic clarity. Finally, revision and re-coding, where necessary, were done. The process was intentionally flexible and cyclical rather than strictly linear, allowing themes to evolve naturally while maintaining methodological transparency and rigor among the researchers.

### 2.6 Ethical statement

This study was conducted in accordance with the ethical principles outlined in the Declaration of Helsinki. Ethical approval was obtained from the ethics committees of the School of Medicine, University of Belgrade (No. 27/V-1) and the Institute for Public Health of Serbia “Dr Milan Jovanović Batut” (No. 2827/1). FG participants were informed about the purpose and procedures of the study, both in written and oral form. They were provided with printed informed consent forms, which they signed before participation. All participants were explicitly reminded—both prior to and during the FGs—that their involvement was voluntary, and that they were free to withdraw from the discussion at any time, without providing a reason and without any consequences. Anonymity and confidentiality were ensured throughout the research process, and all data were securely stored and handled in compliance with European (General Data Protection Regulation No. 679/2016) and national (The Official Gazette of the Republic of Serbia No. 87/2018) data protection guidelines.

## 3 Results

### 3.1 Radiology community

#### 3.1.1 On AI and AI in medicine

A small number of participants answered the question related to how they would define AI. They stated that AI can be described as “*a sort of software solution, which, through combinations of various data entered into the system and different kinds of examples, can come to some conclusions by processing the data and by comparing different types of data and everything*” (G1R2), or as a “*production model”* that “*uses some data as input and makes predictions.”* (G2R7). Elicited by the moderator, some others joined the discussion and later agreed that AI is a branch of computer science that deals with the development of systems and programs for solving problems previously considered to require human intelligence to solve. All participants unanimously agreed that AI cannot replace human intelligence: it can only help people in their work with human guidance and supervision. The use of AI can reduce the time required to complete a task while minimizing the chance of human errors. AI is a tool, and how it will be used depends on people: it is “*a matter of politics,”* that is, “*a question of how we organize ourselves in society*” (G2R7).

The participants believe that AI has the potential to be applied in numerous medical fields, including pathology, surgery, and gynecology, and, as almost all emphasized, especially in radiology. In more general terms, they recognize its applicability “*anywhere where there is a quantifiable parameter*” (G2R2), for “*complete analysis,*” for “*everything, from start to finish*” (G1R2) (Table 1).

**Table 1:** Proposed examples of AI application in medicine.

Medical applications of AI
Medical history-taking
Interpretation of diagnostic imaging under physician supervision
Interpretation of laboratory/biochemical values
Treatment algorithm suggestion

#### 3.1.2 On AI in BCS

Two participants confirmed some experience with opportunistic screening, three prior involvement in organized screening, while one said that this type of examination is not conducted at their clinic. Only one participant—a screening program coordinator for her hospital—reported familiarity with organized screening. She shared that in Serbia, such programs exist for cervical, colorectal, and breast cancer. Other participants did not answer the question about organized screening and spoke instead about screening in general. When asked, they recognized several shortcomings in screening programs in Serbia, the most pronounced being: insufficient system capacity for invitation send-out and cancer screening test conduction, poor organization, and long waiting times.

“I mean, you can wait for the screening, but the process alone makes people feel discouraged so they often wonder, “what's in it for me?”.” (G2R1)

One participant believes that a unified register of patient information would enable the selection of high-risk patients to be prioritized for screening. “*It would be much better if we made a sort of classification of people who would benefit from these tests,*” as he pointed out (G2R7).

Participants also identified the problem of physicians in health centers, especially in small towns, not informing patients about screening. This is interpreted as part of the broader problem of a lack of doctor-patient communication, which they consider to be the result of healthcare system overload. Distributing brochures with screening-related information was proposed as a solution.

BCS was discussed by only three female participants who are part of organized screening programs. They said that the Program works by inviting women of the appropriate age, registered at their health center, for a mammogram every two years, praising excellent response rates. Two of the participants indicated that women are invited by phone. The first readers of mammograms are their radiologists, and in cases where cancer is suspected, a breast ultrasound and, if necessary, a biopsy are performed, or the biopsy is scheduled immediately, i.e., as soon as possible, and with a maximum delay of three weeks, as reported by one participant. In her institution, located in a big city, independent double mammography reading is done, and if there are any discrepancies, a third reader is introduced. Another participant, who works in a small town with an insufficient number of radiologists, and is currently in training to become a supervisor, explained that they perform single-reading mammography only. They usually perform the readings at night and often on Sundays because they lack adequate conditions on workdays, she added.

Participants believe that AI in mammography could play the role of one of the mammogram readers with human supervision. AI would be “*just another reader in a row*” (G1R9), i.e., “*replacing one reader*” (G2R7). Some believe that it could be used to “*follow-up on suspicious areas identified in the scan with a second reading, or a third reading*” (G2R1). In their opinion, AI could be used to mark suspicious scans or parts of the scans, “*as an alarm to prevent missing something,*” “*that maybe something needs to be checked once more*” (G1R2), to draw physicians' attention to elements that could be overlooked due to crowding, fatigue, lack of concentration, work overload. Participants also note that AI could be useful for resolving radiologists' doubts that sometimes arise, no matter the years of experience, or as a confirmation of their expert opinion, acting as the fourth reader, i.e., a kind of “*assurance*” (G1R2). The role of AI as the first reader and its application in triage systems is also mentioned, as it is far easier to “*train the model to reject if there is nothing, than to tell you, here is an irregularity*” (G2R7). One participant specified that a radiologist would be able to classify the irregularities marked by AI. In comparison, another participant emphasized the usefulness of AI in evaluating dense breasts. In addition to mammogram reading, participants identified other uses of AI in mammography: data collection, risk stratification among screening invitees, and screening invitation.

Participants would use AI at work to “*facilitate and accelerate*” the workflow (G1R9), as it would be “*a great help*” (G1R3), and because it would improve work quality due to “*double control*” (G1R3) and by reducing potential human errors (fatigue, subjectivity). AI could “*draw physicians' attention to something that might help them in the rush*” (G1R4) and help them solve dilemmas. The use of AI would allow participants to feel more confident in their work; however, they would always prioritize their own opinion. In cases of doubt or uncertainty, they would use both the help of AI and that of their colleagues. The exception to this is a participant, Program coordinator at a big city health center, with five other radiologists, who stated that the help of a colleague means more to her. They also stated that AI could help train young specialists and residents, given the insufficient number of radiologists.

Participants are “*willing to accept AI help*’*, but not to blindly trust it.*” (G1R7). This expressed lack of trust in AI stems not only from radiologists' beliefs and attitudes but also from its current inadequacy in handling more complex cases, as they noted.

“If we scratch the surface, see how it functions, we see that each complex case in a tertiary institution—there's no way AI'll be dealing with that any time soon. Minor issues—that can be solved, but it's routinely time-consuming for humans anyway. Or, let's say, in New Zealand, I know they were using AI in the ER for pulmonary embolism screening. That can perform quite accurately. To mark suspicious images, even non-contrast. So, in that case, it can improve. We’ll see how it evolves. Similar minor issues—detection of round nodules—that would be workable. But pancreatic cancer staging—now, that would be difficult”. (G2R7)

Radiologists identified the following requirements to trust AI: scientific validation of its efficacy, assessment by an independent body, and personal testing “*through thousands and thousands of mammograms*” (G1R3). Even with these criteria met, they would never completely trust AI and always prioritize their opinions, as stated earlier. Given their belief that AI cannot be made error-free, they would fear inaccuracy while using it in their workplace. Another cause for concern would be the possible corruption of the designated assessment body and its false positive evaluation of AI, which would allow use without “*specified performances*” (G2R7).

“I wouldn't sign the AI results. I would always listen to myself. (…) In case I think something is certainly benign, I would run it, just to see if AI shares my opinion. (…) Anyhow, in cases where I'm completely sure, I could say, let's see if AI interprets it as negative, too. Yes, I would do that. But if I think it's positive… I would probably even use AI to test it. But my opinion would be decisive”. (G1R3)

### 3.2 Screening-aged women

#### 3.2.1 On BCS

Half of the participants have experience with mammography and undergo this type of examination regularly. Most of them describe it as painful and unpleasant, regardless of the place where it was performed (public or private institution, health center, specialist clinic, or mobile mammography unit)—“*painful is painful*” (G1S4). The difference in physical sensations during imaging is made by the speed and skill of the medical staff performing the examination, and above all by their experience. Psychologically, the mammography experience is affected by the staff's attitude towards the patients. In this regard, participants had varied experiences in different places unrelated to the category of the institution in question.

Some participants observed that it is quite difficult to schedule a mammogram at a public institution, that it is a very “*long and unpleasant process,*” “*that the part of the system in charge of it is against you,*” and that it “*does not allow preventive examinations*” (G1S3). They also remarked that patients who want to go to preventative examinations i.e., check-ups are treated as “*people with mental health conditions who are looking for something that isn't there and are bored*” (G1S3), and that physicians then, if the disease is diagnosed (at an advanced stage), “*say that you are too late*” (G1S8), “*where you have been and why hadn't you come earlier?*” (G1S3). Some suggested that scheduling an examination and getting results in the private healthcare sector is simply faster.

Three participants had cases of breast cancer in their families, and 12 recounted the diagnosis in their immediate environments. Breast cancer in their social circles was by far the most common motivation for mammography, often coupled with additional reasons, while some participants identified several other individual reasons to undergo this exam (Table 2).

**Table 2:** Reasons to undergo mammography.

Singular per-case reasons	Reasons in addition to a breast cancer diagnosis in the immediate environment
Genetic predisposition	A lower price for a general check-up (including mammography) compared to the sum of individual exam costs
Breast pain	Suspicious signs (breast pain, a problematic breast mole)
Undergoing regular preventive examinations in general	Media exposure of the significance of mammography
Gynecologist's referral after the age of 50	Get expert “*clean bill of health*”

Most participants felt fear, anxiety, and uncertainty following mammography. Upon receiving the results, the anxiety of waiting was replaced by relief and happiness. Two participants admonished that, despite the fear, “*prevention is better than cure*” (G2S8), while one participant “*didn't have any particular feelings*” because she “*had no specific problems, so that she didn't even think that something could go wrong*” (G2S4).

Participants who did not undergo mammography reported the following causes (Table 3).

**Table 3:** Reasons for deferring mammography.

Reasons
Avoiding unnecessary radiation exposure Absence of known genetic risk factors Absence of medical conditions Age recommendation perceptions Absence of suspicion at breast ultrasound/palpation by gynecologist Prospective pregnancy Public healthcare system shortcomings and a lack of financial resources to undergo mammography privately Focus on confirmed medical conditions (see G.)

Most of these participants omitted answering whether they are familiar with the scheduling procedure for this type of exam, while a few answered in the negative. The exception was a participant whose friend informed her that getting a mammogram at the health center without prior referral was possible by applying for an appointment after the Program announcement. The rest concurred that they would search for scheduling information online and inquire among physician friends for public healthcare, or call to make an appointment at a private clinic.

These participants could be motivated to undergo mammography by being invited to a pre-scheduled exam, either individually or through organized BCS. Participants shared it is generally challenging to get a public healthcare appointment, a time and energy-consuming process, as well as that “*they don't have room to devote to preventive examinations in the system as it currently is*”*,* nor are they willing to pay for private healthcare when “*they are constantly paying for health insurance from their salary*” (G1S6). They added “*it would certainly be easier to get a mammogram, just that it is almost never included in the offer*” (G1S8), as well as that they need someone to “*give them no choice*” but to go (G2S3).

Participants believe that the risk factors for breast cancer development include: a genetic predisposition, age (over 35), and disregard for preventative exams. Some, however, maintain there are no rules as to who will develop the disease. Concerning genetic predisposition, they suggested preventive examinations start at an earlier age and be performed more often, and underscored dietary changes as significant. For women at risk of breast cancer, education and organized BCS, with potential penalties in cases of non-attendance, would be important, as they noted.

Generally, participants are not fully familiar with the details of mammogram reading. Two opinions are equally represented as dominant, the first being that a mammogram is read by several radiologists if suspicious of irregularity, otherwise it is done by one; and the second being that it is read by several radiologists always. Two participants stated that a mammogram is read by the radiologist, without additional explanation, while two conceded they are unfamiliar with it. A participant shared that one doctor read her scan, while another said that, in her case, due to suspected malignancy, three radiologists did so.

The majority of participants trust mammogram readings, emphasizing the importance of a physician's professionalism, competence, and prior acquaintance. The perfect scenario implies physician selection for mammogram reading, which they consider a privilege usually associated with the private healthcare sector. Two participants stated that they have more trust in public healthcare, while another two noted that their trust is contingent on the doctor doing the reading. Trust in a physician depends not only on his knowledge and expertise but also on his approach to the patient, as some pointed out. Trust is very important and difficult to gain, as another one posited, which is why she believes that one shouldn't change good doctors, even if it means that they need to pay for examinations, should they relocate to private practice. Finally, a participant concluded that “*they have to trust wherever they go*” (G2S8) if they cannot choose.

#### 3.2.2 On AI in BCS

Most participants would not trust the mammogram reading performed by AI, as they exclusively trust physicians. They believe that AI is prone to errors, malfunctions—“*every machine, device breaks down*” (G1S1). The reason for mistrust lies in the fact that AI development is still in progress, and there exists a general concern about its reliability, as they stated.

“I don't know how accurate it can be. How reliable it can be in terms of accuracy. Precisely because someone with years of experience can recognize and know it. I think the robot can recognize the change, but the question is how well it can define it in the right way and make that definition reliable and accurate. It may say that it can see the change, but say that it is adipose tissue, a sebaceous gland, a mammary gland, something benign. It's something I can't believe.” (G2S8)

Participants prefer the mammogram reading done by a physician over AI because the physician “has emotions and empathy and is somehow… human” (G1S1), “also has digital literacy, as well as social and economic literacy, and so on,” that is, “he simply passed the exam”, and the patient can guide the physician in “navigation” during the exam (G1S2). Participants also mentioned that the use of AI discriminates against people, and that people feel like “lab rats for a very long time in many fields” (G1S3).

Few participants believe that both a physician and AI could read a mammogram, but with the supervision of a doctor, whose opinion would be final. As one participant explained, AI would be useful for detecting and marking changes while the radiologist would interpret such changes.

“AI, if data from a large number of reference institutions has been entered into the AI system—you know, there are clinics, both German clinics and Chinese clinics and the ones that, with all due respect, are three times better and more experienced, which have a larger volume of work than ours—then I would trust AI for physical characteristics, for measuring millimeters, milligrams, when we talk about scans. And subjectively, whether the black tissue is a thread, a string, or healthy tissue, I would perhaps trust a human more. So yes, I vote for the mix, if possible, all the while knowing who corrected the AI. I mean, I would like to know the radiologist's background.” (G1S3)

The upside of such AI use in mammogram reading would be “*to speed it up to some extent, maybe in that sense, and then there will be no more waits and anything*” (G2S7). One participant believes that AI could be used as an aid in scan reading but remarked that it will never be able to replace the “*health professionals' communication,*” “*human approach,*” because “*if you don't open up, if you can't be relaxed in front of someone who examines you, you can skip something, and simply navigate to something else. To put it simply, a machine is a machine, and a man is a man.*” (G2S5)

Participants also pointed out that AI is something “*top notch,*” but Serbia still has not reached the “*elementary level*” (G1S6) that should be addressed first. This includes the procurement of good diagnostic equipment, because the existing equipment is in poor condition and constantly breaks down, as they stated, and other basic resources. Facilitated access to appointment-making and examinations should also be made available. One participant explained that due to the mentioned problems, the use of AI in diagnostics is “*beyond the interest of us ordinary people*” (G1S6).

Participants' opinions on the application of AI in screening and medicine are split. Three participants omitted answering this question; three of them saw no room for the use of AI in medicine, and the rest identified several such uses (Table 4). Most believe that the use of diagnostic AI in Serbia would be endorsed if the results were visible and trust gained, as was the case with other past innovations, as one participant pointed out.

**Table 4:** Potential AI uses in mammography (women's perspectives).

Uses
Mammogram reading
Data entry and analysis
Selection of patients at risk
Administration (e.g., inviting patients for appointments)

## 4 Discussion

Radiologists believe that AI has the potential to be applied in medicine, primarily in radiology. Radiology is also viewed as a healthcare specialty most linked to AI use in a qualitative study conducted among physicians in Turkey (Kahraman et al., 2024). In mammography, the radiologists from our study see the possibility for AI application in mammogram interpretation under human supervision. Existing studies purport that radiologists do not want AI to replace all human readers, with a cautious disinclination towards scenarios where AI yields greater autonomy, seeing it instead as a means to increase their sensitivity for cancer detection (46, 49–51). Similar to other studies, radiologists in Serbia believe that AI could be a useful tool in their workplace, which would shorten interpretation times, speed up the workflow, and reduce the probability of errors (46, 49–51). In addition to mammogram reading, radiology specialists in Serbia believe that AI could also be used for data collection and for patient invitation to mammography BCS. These non-diagnostic tasks, among others, have the potential to optimize workflow, improve its quality and efficiency, as well as to improve patient satisfaction (52). Participants also believe that one of the applications of AI in mammography could be the selection of patients to be invited for examination by risk evaluation. This form of AI application in mammography has proven to be successful in terms of workload reduction for radiologists, whilst improving early detection rates for breast cancer (53–56). Studies have shown that new risk assessment models using AI show superior performance in predictive accuracy compared to commonly used models (57–59). These studies also suggest that a personalized approach to BCS allows for better, personalized treatment. A study by Braithwaite et al. (60) demonstrates that ChatGPT could be a useful aid for healthcare professionals in designing individual recommendations for BCS in patients over 75, but physician supervision remains necessary due to the software's limitations. Evidence on the utility of AI in diagnostic radiological image interpretation in low and middle-income countries (LMICs) confirms consistent gains in sensitivity, specificity, and applicability across modalities, including ultrasound, x-ray, and computed tomography (61). Improvements in workflow efficiency and support for non-specialist providers are also noted.

When discussing the support for AI use in triage systems in our study, the issue of responsibility for removing cases from the workflow was largely not touched upon, as was the case in some other studies. For example, a survey conducted in Australia showed that most surveyed radiologists would not feel comfortable if made to take responsibility for the use of AI in patient triage, and that responsibility is an omnipresent concern (51). This complex issue did not emerge as a topic in the FGs, except for a couple of participants who pointed out that AI would not be able to replace radiologists, given that someone will have to account for potential software errors as its supervisor. A web-based survey among physicians and medical students in Oman established that half of the respondents believe that both the software manufacturer and the physician should bear legal responsibility for such errors, while more than a third believe that physicians, as the ultimate decision-makers, should be held legally responsible (62). According to the same survey, older respondents showed a lower level of familiarity with AI and greater concern about legal responsibility in case of errors. A EuroAIM survey from 2019 demonstrated that members of the European Society of Radiology equally support the view that responsibility for AI-generated outcomes should rest with radiologists or be shared with other professionals, like AI developers or insurance companies (63). In the literature, human-in-the-loop (HITL) systems are estimated as likely the most common method for integrating AI into clinical practices (64). HILT systems “combine human expertise with AI, allowing a collaborative decision-making process that increases diagnostic accuracy and efficiency” (64, p. 5). This means that the question of responsibility for errors from AI use in mammography is complex and should be shared among all the stakeholders involved. In HILT systems, radiologists likely maintain primary responsibility for interpreting AI-generated results and making clinical decisions. However, AI developers must also ensure that their algorithms are accurate and reliable. Precisely defining the responsibility of experts by law in relation to the provision of services performed independently by AI, balancing between human control and technical autonomy, will be necessary, as Neri et al. (65) noted. The radiologists in our study did not reflect on the unclear responsibility for algorithm errors, risk to privacy and data security, or other ethical issues and principles such as informed consent, human rights and dignity, justice/fairness/equality, integrity/honesty, generally recognized in the literature (64, 66–75). Analyzing the traditional legal frame's adaptability in the field of medicine and healthcare to new AI technologies and medical tools in the context of various ethical issues, and especially in terms of application safety, Nikolić Popadić and Sjeničić (76) concluded that it is necessary to modify the existing legislation and/or adopt new laws, in EU and in Serbia, to keep up with the fast-paced innovations in this field.

Radiologists in our study are keen on using AI at the workplace: they perceive AI as an aid that would improve and speed up their work, owing to the possibility of training young specialists and residents, and due to the insufficient number of radiologists on staff. Favorable outlook on AI adoption in radiology is in line with the results of studies from Europe (49, 63, 77), other LMICs (78–87), and other parts of the world (88, 89, 122). On the other hand, some studies conducted in LMICs report a moderately positive perception of AI use in radiology, with a significant positive correlation between AI literacy and perception (90–92), with the latter being observed in other studies as well (78, 82, 93, 94). Concerning the neighboring countries, Malkić et al. (83) conducted research in Bosnia and Herzegovina among healthcare workers, including radiologists, to explore attitudes towards the acceptance of AI in radiology. Their respondents mostly agree that AI will improve the efficiency of radiological services and treatment processes. The only similar study of this kind conducted in Serbia was among medical students, and it highlighted an overall positive perception of the application of AI in healthcare (95). Our study shows that radiology specialists and residents would feel more confident with the use of AI, but they would always prioritize their own opinion.

Radiologists' lack of trust in AI, observed among our participants, was identified as a possible challenge in this AI application field in other studies, mostly concerning other LMICs (80, 92, 96), but also in high-income countries (HICs) as well, albeit to a lesser extent [e.g., (50, 97)]. In LMICs, it is noted that radiologists, although aware of the benefits of AI application in radiology and willing to use it in their workplace, do not trust AI-generated reports and demand mandatory human oversight over them. This cautious optimism and low confidence in AI tools in LMICs are followed by several other barriers that hamper trust and AI adoption in radiology, such as a lack of formal AI training, concerns about reliability, and legal accountability, non-transparent AI algorithms, health institutions' limited participation in AI production and validation, as identified in the mentioned studies. In LMICs, limited training and infrastructure impede the adoption of AI in radiology compared to HICs, emphasizing the importance of integrating AI into radiology curricula, pilot programs, and regulatory frameworks to navigate these LMIC-specific constraints (91, 92). Similar conclusions follow from research conducted in Bosnia and Herzegovina, where healthcare workers, including radiologists, acknowledge that the application of AI in radiology requires additional education and training (83). The majority of participants declare that they do not feel safe when using AI, suggesting a lack of training and education, and the necessity of overcoming this barrier. Most of them do not agree that the use of AI in radiology can improve the quality of healthcare. This refers to the need for a better understanding of the contribution of AI to the quality of healthcare services among Bosnian radiologists. In HICs, a lack of trust, awareness, knowledge, and practice has been identified as an impediment to the application of AI in radiology among its practitioners, alongside AI being perceived as a threat to radiologists' professional autonomy (123). Some studies report that more broadly, among physicians and medical students, the willingness to use AI in medical practice is dependent on their involvement in developing international guidelines, prior formal training in AI use, and confirmation of AI efficiency by studies published in reputable periodicals (62). Physicians also emphasize the importance of accreditation and regulation of AI systems by scientific societies, and to remain at all times open to global audit performed by impartial, supranational parties (72).

In accordance with the conclusions from the literature above, concerns shared by radiologists in our study can be attributed to a lack of concrete policies in Serbia related to the use of AI in the medical and healthcare field, among other factors. In December 2019, the Government adopted the Strategy for the Development of Artificial Intelligence in the Republic of Serbia for the Period 2020–2025, aligned with the European Artificial Intelligence Initiative (98). The Strategy defined objectives and measures for the development of AI, whose implementation should enable safe development and application of AI, consistent with internationally recognized ethical principles, to utilize its potential to improve the quality of life of each individual and the society as a whole [(76), pp. 29–30]. At the beginning of 2025, the Government adopted a new Strategy for the Development of Artificial Intelligence for the Period 2025–2030, which defines new goals and measures for the continued development and implementation of AI in the country (99). Unlike the previous Strategy, the new one emphasizes the importance of ensuring that artificial intelligence is reliable and responsible. In 2023, the Ethical Guidelines for the Development, Implementation, and Use of Reliable and Responsible Artificial Intelligence were adopted, incorporating principles from UNESCO's 2021 Recommendation on the Ethics of Artificial Intelligence (99, 118). Although not legally binding, they serve as a blueprint for all stakeholders in the AI landscape (99). The Artificial Intelligence Act of the Republic of Serbia has not yet been adopted. The Ministry of Science, Technological Development, and Innovation of the Republic of Serbia has established a working group for drafting the Act, using the EU AI Act as an exemplar (100). Nevertheless, none of these legislations regulates AI applications in healthcare and medicine. The same applies to current legislation in this field in Serbia, such as the Health Care Law, the Law on Patients' Rights, and the Rulebook on the detailed conditions and method of performing the assessment of healthcare technologies (101, 119, 120).

The topic of screening generated a discussion among radiology specialists and residents about the numerous problems in this field in Serbia, as reported. The mentioned problems include the insufficient system capacity for invitation send-out and examinations, poor organization, and long screening waits. The lack of physician-patient communication, a side-effect of healthcare system overload, is also seen as a problem, often resulting in patients being uninformed about screening altogether. The Program's implementation in urban areas is much better than in small towns, where it is performed with difficulty due to inadequate working conditions and insufficient staff. Complementary to that, most screening-aged women agreed that it is challenging to schedule mammography in public institutions and that health-aware patients frequenting preventive examinations are rarely looked upon favorably. Some women added that the problems in public healthcare, as remarked above by radiologists, were among the reasons they had not undergone mammography. An invitation to a scheduled mammogram, individually or as part of an organized BCS effort, was singled out as an important motive to undergo mammography among women who have not yet undergone this type of exam. Consistent with these results from our study are the results obtained from other LMICs. A literature review conducted by Janatolmakan et al. (102) lists several causes of delayed breast cancer diagnosis in LMICs, most of which concern the situation described by our participants, such as problems with various aspects of access (accessibility, availability, acceptability, affordability, and accommodation). Similar to the situation in the public healthcare sector in Serbia, they report the negative perception and reluctance of patients towards receiving services from government hospitals, patients’ dissatisfaction with the lack of empathy from service providers, negative personal experiences with treatment and diagnostic centers from previous visits, inappropriate scheduling, and extended waiting times for patients to undergo primary diagnostic procedures.

Screening-aged women generally would not trust the mammogram reading performed by AI, and most of them believe that mammograms should be read exclusively by radiologists, even without AI assistance. This is discordant with other studies' results, where in a number of different countries participants displayed a positive attitude towards the use of AI in mammography interpretation (43, 45, 47, 48, 93, 94, 103, 104). One such study from Norway revealed that the preferred mammogram reading strategy implies the participation of a single radiologist and AI (94). The prerogative of maintaining human control in the reading process was also noted among screening-aged women in Australia (47, 48). Independent AI use in mammography without radiologist supervision is also not supported by Dutch women, aged 16–75, who preferred the scenario in which the radiologist does the first reading followed by a second reading performed by AI (105). A study from Italy conducted among BCS program participants has yielded the same results (43). In the UK, screening-aged women showed the most support for the possibility of AI being used as a mammogram reading aid or a replacement for one reader, and the least for it being used as a tool in the triage system (93). They neither approved nor rejected the total replacement of human readers. More broadly, a survey conducted in the general population of adult women in England asserted that women of mammography screening age are more likely to have a positive attitude towards the use of AI in BCS (104). In Sweden, a study has shown that as many as 38% of 2,196 participants expressed a preference for an exclusively computerized examination of this kind (103). The highest level of trust was given to the computer reading, followed by the examination by a physician. More data from Serbia and the region on a sample of screening-aged women, or women in general, are unavailable due to the absence of such research; therefore, such comparisons cannot be made. This also applies to research from other LMICs. Concerning the patients’ perspective on the use of AI in radiology not related to BCS, a study from Zimbabwe reveals predominantly negative attitudes and low trust in AI technologies, alongside a low level of knowledge about AI, even though they are aware of the potential benefits, such as shorter wait times (106). This study highlights the demand for greater education, transparent communication from healthcare providers, and ethical guidelines for AI implementation.

Women in our study trust in mammogram readings performed by a radiologist, although they are generally not familiar with the details of this procedure. Ideally, they would like be able to choose which radiologist reads their mammogram. Trust in the radiologist in question is crucial and is based not only on their expertise and competence, but also on their patient approach. The reason for mistrusting AI lies in the belief that it is error-and-malfunction-prone and has still not been perfected. Participants primarily and predominantly give priority to the mammogram interpretation done by a physician, also because of “*human contact*”, which they consider an essential component of the medical examination. Personal interaction with the physician was emphasized as very important in other studies conducted in developed, mostly Western countries [(105, 107, 108); see also (43, 44)]. These studies have shown that patients emphasize the importance of direct communication with the physician, even in the absence of contact with the radiologist reading the mammogram. A lack of empathy has also been reported as a concern when using AI in radiology (109). However, the use of AI in medicine could lead to the strengthening of the physician-patient relationship, and not to its disruption [(110), see also (111)]. With AI taking over routine and repetitive tasks, such as administration, documentation, protocols, and analyses, physicians would have more time to talk with patients, make joint clinical decisions, and provide information, emotional support, and empathy. The ESR members' survey (63) demonstrated that most respondents believe AI will make the radiologist-patient relationship more interactive, while the potential excess time would, in the first place, serve to communicate with clinicians, and secondly, with patients.

A few women in our study believe that mammogram reading could be performed by both the radiologist and AI, but under the radiologist's supervision, whose opinion would be final, which is in line with the preferred scenario in most of the previously mentioned studies. As a benefit of such AI application, they predicted shorter wait times, as in other similar studies [e.g., (108)]. The application of AI in diagnostics is viewed as a feature of the highest level of development, and our participants perceive that the basic conditions, such as good diagnostic equipment and easier mammography access, have not yet been met in Serbia. For these reasons, they believe that this fundamental level should be ensured first, and they do not currently consider diagnostic use of AI a topic of interest.

Among other uses, women in our study identified mammogram reading as a pliable point AI introduction (Table 4). Although they remain skeptical, most participants believe this application would garner support in Serbia if its results were satisfactory and due trust earned. A study in Sweden has shown screening-aged women's trust in AI requires thorough evaluation, transparency regarding AI use in healthcare, and radiologist involvement in the assessment process (45). Another survey in England found that this trust was assessed as low, and dependent on the following: thorough research, gradual introduction, double-checking mammography readings, data protection and security, and continuous human presence (112). Support for the use of AI in mammography in Australian women also calls for strong evidence of system performance, sufficiently clear and convincing explanations for AI introduction, and familiarity with AI (48). To achieve the latter, three solutions have been proposed: transparency and provision of information, slow and gradual introduction, and the possibility for women to choose whether AI analyzes their mammograms or not. Another study conducted in Australia highlighted expectations of clear delineation of decision-making responsibilities between AI and human supervisors, the ability to challenge decisions made by AI, and that an AI-based system should work equally well for all participants in the BCS program (47).

## 5 Strengths and limitations

To our knowledge, this is the first study in Serbia that deals with the perceptions of AI use in mammography screening among radiologists and screening-aged women. Research has shown that the implementation of AI-based software in medicine largely depends on the attitudes of both physicians and patients, and this applies to mammography screening as well (44, 48, 64, 108, 109), while the effectiveness of the use of AI in this field and beyond has been documented before, as discussed above. Taking into account the situation in Serbia—an insufficient number of radiologists, a large number of women of mammography-recommended age, and high breast cancer morbidity and mortality rates (113–116)—the results of this study can be used for more straightforward future implementation of AI in BCS and for overcoming the potential barriers on that path. Under such circumstances, the application of AI would be extremely important for achieving greater screening coverage, improving prevention, speeding up the workflow, and reducing the radiologists' workload. Another strength of our study is the overall high knowledge level among radiologists recruited to participate in the specialist FG, compared to the average. Participants were recruited during continuous medical education for mammography screening specifically, which makes their answers extremely relevant in the mentioned context.

However, this study has certain limitations. As with all qualitative research, findings are not intended to be statistically generalizable. Not all stakeholder groups were included in our study for logistic reasons (radiographers being the most noteworthy), and not all viewpoints were represented, despite having used purposive sampling with maximum variation (women from a rural background were underrepresented). Group dynamics may have influenced the expression of individual views despite active moderation. Radiology specialists and residents were reserved in the FG discussions overall, and only a few participants offered the most answers in both groups. The number of these participants per group was below one-third, exceeding that margin only on a select few topics, rarely surpassing half. Given the lack of studies on this topic in Serbia, potential explanations for this phenomenon cannot be extracted from existing literature. However, the very absence of such studies suggests that in the given context, there is not enough interest in the topic of AI use in medicine still. The restraint and possible disinterest among radiologists included in our study could also be interpreted as a side effect of work overload and the working conditions, generally perceived as inadequate among physicians employed in the public sector, as was the case with all FG participants, which is why they do not consider this topic a current priority. In addition, the Specialty and Subspecialty Rulebook for Healthcare Workers and Healthcare Associates (117) stipulates that breast radiology is covered in the 4th year of radiology residency, which is why most participants in the resident FG may not have felt confident enough to join the discussion, being second and third years. The above explanations should be considered with reservations, and the described situation should be taken as an incentive for additional research on the same sample and beyond.

## 6 Concluding remarks

This study represents the first step towards understanding the attitudes of radiologists and screening-aged women in Serbia towards the use of AI in mammography BCS. Radiologists see room for and have an overall cautiously positive attitude toward the application of AI in mammography BCS in the future. Also, our study suggests that the use of AI in mammography BCS could be met with support among women if AI were to perceptibly improve the current state of healthcare, which remains to be seen.

Additional studies will be necessary to get a more comprehensive overview, especially given the limitations listed above. Future analyses of the attitudes of target population women in Serbia should include an investigation of knowledge levels on AI, shown to be an important predictor of AI acceptance.

## Data Availability

The raw data supporting the conclusions of this article will be made available by the authors, without undue reservation.
